# A Contact-Imaging Based Microfluidic Cytometer with Machine-Learning for Single-Frame Super-Resolution Processing

**DOI:** 10.1371/journal.pone.0104539

**Published:** 2014-08-11

**Authors:** Xiwei Huang, Jinhong Guo, Xiaolong Wang, Mei Yan, Yuejun Kang, Hao Yu

**Affiliations:** 1 School of Electrical and Electronic Engineering, Nanyang Technological University, Singapore, Singapore; 2 School of Chemical and Biomedical Engineering, Nanyang Technological University, Singapore, Singapore; Dalhousie University, Canada

## Abstract

Lensless microfluidic imaging with super-resolution processing has become a promising solution to miniaturize the conventional flow cytometer for point-of-care applications. The previous multi-frame super-resolution processing system can improve resolution but has limited cell flow rate and hence low throughput when capturing multiple subpixel-shifted cell images. This paper introduces a single-frame super-resolution processing with on-line machine-learning for contact images of cells. A corresponding contact-imaging based microfluidic cytometer prototype is demonstrated for cell recognition and counting. Compared with commercial flow cytometer, less than 8% error is observed for absolute number of microbeads; and 0.10 coefficient of variation is observed for cell-ratio of mixed RBC and HepG2 cells in solution.

## Introduction

Flow cytometer has been widely deployed in biological research and clinical diagnostics to automatically determine the count or concentration for one or multiple types of cells [1]–[4]. For example, in HIV monitoring and treatment, counting of CD4+ and CD8+ T-lymphocytes are required for antiretroviral therapy [5]. In immunophenotyping, human peripheral blood samples are analyzed by calculating cell concentrations for platelets, lymphocytes, and monocytes [6]. All these applications demand high accuracy and throughput with the use of flow cytometer. A conventional flow cytometer measurement is performed by passing a narrow stream of cells through a focused laser beam at a rate of thousands of cells per second. The optical signals such as forward scattering (FSC), side scattering (SSC), fluorescent light emission (FL) are simultaneously measured to obtain information such as relative size, granularity or internal composition of cells. However, because of the bulky equipment size with sophisticated optical measurement procedure, the conventional flow cytometer is prohibitive for point-of-care application [7]–[8]. In addition, flow cytometry is traditionally relied on non-imaging technique by laser scattering and fluorescence emission for cell identification [9]–[11] and hence is lack of image information of cells [12]–[13].

The recent advance of microfluidics-based lab-on-a-chip technology has introduced the possibility for the miniaturized microflow cytometer for potable flow cytometry [9]–[11], [14]–[15]. With the integration of complementary metal oxide semiconductor (CMOS) image sensor chip underneath the microfluidic channel, microfluidics-based lensless imaging systems [16]–[22] can be developed for portable contact-imaging [23] based microflow cytometer. Illuminated by incoherent light source, the direct projected shadow or contact images of cells can be captured by the image sensor underneath without lenses [16]–[22].

**Figure 1:**
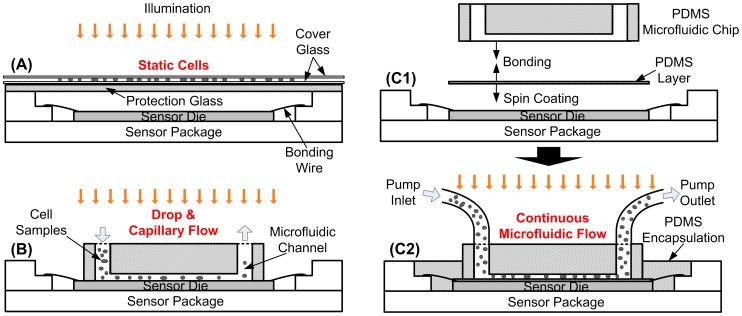
Different contact imaging systems without optical lens. (A) Static contact imaging system. (B) Microfluidic contact imaging system with capillary flow. (C) The proposed microfluidic contact-imaging cytometer system with continuous flow: (C1) bonding process; (C2) overall system structure.

However, the captured images of microfluidic flowing cells are intrinsically in low-resolution (LR) with loss of details in cell morphology information since there is no optical lens for the flowing samples. As shown in Fig. 1(A), one Lensless Ultra wide-field Cell monitoring Array platform based on Shadow imaging (LUCAS) system is demonstrated for cell counting application [19]. To distinguish different cell types, the cell intensity distribution pattern of raw LR shadow or holographic shadow images are used [19], [22]. The cells to be imaged are statically placed in between cover slides above the image sensor array. Thus, without continuously flowing microfluidic, the total solution volume is limited in each test. In [17]–[18], a multi-frame sub-pixel resolving super-resolution (SR) processing is proposed with a high-resolution (HR) cell image recovered by capturing a large number (40 to 100) of subpixel-shifted LR cell images. As shown in Fig. 1(B), in order to capture subpixel motions in multiple frames, a drop-and-flow is employed to maintain the low flowing speed, usually driven by capillary or electroosmotic flow for precise movement control. Moreover, the storage of multiple cell images to recover one SR image consumes huge hardware resource. Both problems limit the throughput when counting multiple continuously flowing cells.

In this article, a contact-imaging based microfluidic cytometer is introduced with extreme-learning-machine based single-frame SR processing (ELM-SR) that can perform recognition and counting of cells in continuously flowing solution. The Extreme Learning Machine (ELM) is a general suite of machine-learning techniques. ELM theories and algorithms have been successfully used in many applications such as bioinformatics, image processing, feature selection, human action recognition, etc. To our best knowledge, this paper represents the first study applying the ELM analysis to achieve single-frame super-resolution for cell imaging. Compared to the single-frame SR by interpolation and sharpening [24] the pattern-recognition based SR [25]–[26] can recover high-frequency (HF) components containing details for fine structures in cells. In addition, with randomly generated weights between input layer and hidden layer, the pattern-recognition based SR in this paper is based on extreme learning machine (ELM) that can have much less expensive iterative training process for on-line SR image recovering [27]. Here is the flow of the developed single-frame ELM-SR for the contact-imaging based microfluidic cytometer. Static HR cell images obtained from microscope are first classified and stored as off-line HR cell image library, which are utilized to train an ELM-SR reference model. Note that HR cell images in library contain the detailed internal cell structure information with HF components. Then, the on-line single-frame SR processing is performed by employing the ELM-SR reference model to recover the necessary HF components from one on-line LR cell image. The recognition and counting for different types of flowing cells can be thereby performed accurately by only checking for the strongest structure similarity [28] with reference to the off-line static HR images. As such, the developed microfluidic imaging cytometer can achieve single-cell image quality without flow rate limitation when compared with [17]–[18].

We examined the performance of the prototype of the microfluidic cytometer by measuring the absolute number of microbeads in solution per unit time of flow, and the concentration ratio of mixed flowing HepG2 and red-blood cells (RBCs) both at a flow-rate of 5 µL/min. Less than 8% error is observed for the absolute number of microbeads; and a coefficient variation of 0.10 is observed for the cell ratio when compared with a commercial flow cytometer. The demonstrated microfluidic imaging cytometer is thereby meaningful for rapid counting of various cells for point-of-care diagnosis as well as for water quality analysis in remote and resource-limited areas.

## Materials and Methods

### System Overview

The proposed contact-imaging based microfluidic cytometer for flowing cell recognition and counting is shown in Fig. 1(C). It includes one PDMS microfluidic channel attached on top of a CMOS image sensor, through which cells flow continuously. A syringe pump continuously drives the sample solution of interest into the channel and controls the flow rate. A conventional white LED lamp is applied as the light source above to project flowing microbeads or cells in the solution. The CMOS image sensor can continuously capture shadow images underneath. The captured digital image frames are then rapidly processed with machine-learning based single-frame SR algorithm, which can improve resolution of shadow images such that one can recognize and count the flowing cells.

### Microfluidic Channel Fabrication

The PDMS-based microfluidic channel was fabricated by the conventional soft-lithography [29]. The channel features were designed in AutoCAD (Autodesk, San Rafael, CA) and then written to a transparent mask. Then negative photoresist SU-8 (SU-8 25, Microchem, MA) was spin-coated (SCS G3P-8, Indianapolis, IN) on a 3-inch polished silicon wafer to fabricate the SU-8 mould. Afterwards, a volumetric ratio of 10∶1 mixture of PDMS (Sylgard 184, Dow Corning, MI) and curing agent were poured onto the SU-8 mould. After degassing and curing, the PDMS replica was peeled off from the master and punched on top for inlet and outlet, which were connected with silastic laboratory tubings to syringe pump and waste bin.

### Microbead and Cell Sample Preparation

In the experiment, HepG2 cells (American Type Culture Collection, MD) were cultured in Minimum Essential Media (MEM) (Gibco, cat# 11095-080) supplemented with 10% fetal bovine serum (FBS) (Gibco, cat# 10270-106), 1 mM sodium pyruvate (Gibco, cat# 11360-070), 0.1 mM MEM non-essential amino acids (Gibco, cat# 11140-050), and grown at 37°C under a 5% CO2 atmosphere in a T75 flask. The harvested cells were washed and re-suspended in phosphate-buffered saline (PBS). (Fisher Scientific, Pittsburgh, PA). The RBCs were obtained from National University Hospital (NUH) Singapore, also suspended in PBS. The polystyrene microbeads of 6 µm diameter (Product# 07312, Polysciences, Warrington, PA) was selected for calibration experiments as it is of similar size with RBC. The microbeads were suspended in PBS. All the samples were sonicated in an ultrasonic benchtop cleaner (Branson 2510E-DTH, Danbury, CT) for 10 minutes before pumping into the microfluidic channel to prevent aggregation.

### Microfluidic Cytometer Design

**Figure 2:**
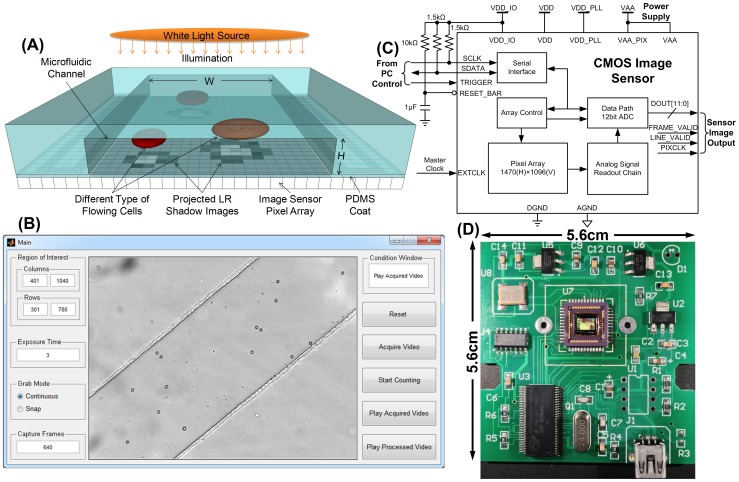
Microfluidic contact-imaging cytometer system for flowing cell detection, recognition and counting. (A) Cell shadow image by contact imaging. (B) Captured video of flowing cells. (C) CMOS image sensor board schematic with external controls. (D) System board of the developed microfluidic cytometer.

To build the contact-imaging based microfluidic cytometer with higher spatial resolution, a grayscale CMOS image sensor (Aptina MT9M032, San Jose, CA) is selected with a pixel size of 2.2 µm×2.2 µm. The active sensing area is 3.24 mm(H)×2.41 mm (V) by a 1472(H)×1096(V) pixel array. The hardware design is shown in Fig. 2(C) and 2(D).

As shown in Fig. 2(A), the developed microfluidic cytometer is based on contact imaging [16]–[22], where the light intensity and contrast of one cell's shadow image is determined by the distance *D_obj_* from the object to the pixel array. Note that shorter object distance *D_obj_* provides better contrast *C_on_* and resolution due to less diffraction effect [23],
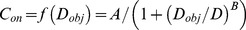
(1)


where *A* is the contrast amplitude, *D* is the characteristic distance, and *B* is the shape parameter. Guided by (1), we first discuss the design of microfluidic channel and then CMOS image sensor.

Firstly, the protection glass of the image sensor chip was first removed before bonding with PDMS microfluidic channel. In addition, the microlens layer above the pixel array is removed by treating the sensor under oxygen plasma (PDC-32G, Harrick Plasma, Ithaca, NY) for 45 min (18 W) [17]. However, as the developed system utilizes the continuous microfluidic flow, which generates higher pressure to the channel wall than the one using capillary or electroosmotic flow [16]–[17], a thin PDMS layer was also spin coated on top of the sensor die. A tight PDMS-PDMS bonding [14] is required as the process shown in Fig. 1(C1). The spin speed of 9000 rpm is set to generate a thickness of 6 µm [30] for PDMS. Therefore, the object distance of our system is 6 µm to enable enough contrast for the microfluidic contact imaging. After spin coating and baking, the surfaces of the microfluidic channel and the image sensor were cleaned with ethanol and oxygen plasma and are further bonded together finally as shown in Fig. 1(C2). Note that after bonding the PDMS coated sensor chip and the microfluidic chip, we also filled the PDMS and curing agent mixture into the gap to encapsulate the bonding wires.

Moreover, to make the full use of the active pixel area, the channel length was designed as 4.6 mm and cut in diagonal. Thus when bonded on top of the sensor die, the rectangle microfluidic chip was just within the die area of the bonding wire. A relative wide channel width of 500 µm was chosen such that high concentration of cells can flow through the channel without clogging [31]. The height of the microfluidic channel was 30 µm, just higher than the normal cell diameters. This ensures that the cells flow close to the sensor surface with better projected image contrast [17]. Besides, in order to improve the wettability of the channel, the channel was coated with bovine serum albumin (BSA) by flowing a 1% solution of BSA in PBS through the channel for an hour [32].

Next, the CMOS image sensor chip was soldered on one low-cost 5.6 cm×5.6 cm printed circuit board (PCB) that provides the sensor with power supplies and digital control signals as shown in Fig. 2(D). The data transferred from the CMOS image sensor to PC was through a USB interface (CY7C68013-56 EZ-USB FX2, San Jose, CA), which ensures high-speed imaging with maximum data transfer rates of 56 Mbytes per second. The sensor working status such as exposure time, ROI and number of frames to capture was controlled by the status registers that can be accessed through a two-wire serial interface, i.e., SCLK and SDATA, as the schematic shown in Fig. 2(C). They are set through the custom designed GUI shown in Fig. 2(B). We set 640×480 image ROI of the sensor to capture the flowing specimens at a sensor frame rate of ∼200 frames/s (fps).

In the experiments, the microfluidic chip was connected to a syringe pump (KDS Legato180, Holliston, MA) through silastic laboratory tubings and samples were pumped into the microfluidic chamber continuously at a typical flow rate of ∼5 µL/min under the illumination from a white light source (Olympus LG-PS2, Tokyo, Japan). The thin tubing of 0.64 mm i.d. and 1.19 mm o.d. (product no. TW-96115-04, Cole-Parmer, Vernon Hills, IL) was used as it helps reduce dead volume and cell lost compared with thick channel. The light source was placed 12 cm above the sensor and the light intensity at the sensor surface was 1.5 k Lux. The exposure time of the sensor was set ∼75 µs, corresponding to 3 rows of sensor readout time. The readout LR frames were buffered with digital image processing conducted to improve the resolution by single-frame ELM-SR processing. As such, the developed system can automatically recognize and count the flowing cells.

### Contact Image Processing of Flowing Cell Frames

Digital image processing is performed to recognize and count the cells flowing through the microfluidic channel. The processing includes three repeating steps to all the captured frames with the flowing cells: 1) temporal-differencing based flowing cell detection [33]–[34]; 2) single-frame ELM-SR based cell type recognition [27]; and 3) cell counting of each type.

#### Temporal-differencing based Flowing Cell Detection

All the flowing cells in each LR frame need to be detected first. This is realized by the temporal-differencing based background subtraction [33]–[34]. Starting from the first two frames, where the first one is the reference (or background) frame and the second is the current (or foreground) frame, moving cell contours in current frame is detected by subtracting it with its previous reference frame to obtain a pixel-by-pixel intensity difference. After subtraction, the regions where the intensity differences are zero indicate no moving cells; and those non-zero difference regions are caused by the motion of cells in the channel, or by the addition and removal of a cell from the sensor field-of-view (FOV). A suitable intensity threshold can be set to identify the contours of moving cells from the background in all frames [33]. The time-difference between each two consecutive frames is determined by the sensor frame rate. Note that each detected cell in one frame will be assigned with one unique identification number, which means the cell count of the current frame.

#### ELM-SR based Flowing Cell Recognition

As the raw detected cell images have low resolution, SR processing needs to be performed for better cell type recognition and further counting. In order to resolve the problems of low flow speed and large storage requirement, the inherent limitations of the previous microfluidic imaging system with multi-frame SR processing [17]–[18], the machine-learning based single-frame SR is developed for the proposed contact-imaging microfluidic cytometer.

*A. ELM-SR Training and Testing*

**Figure 3:**
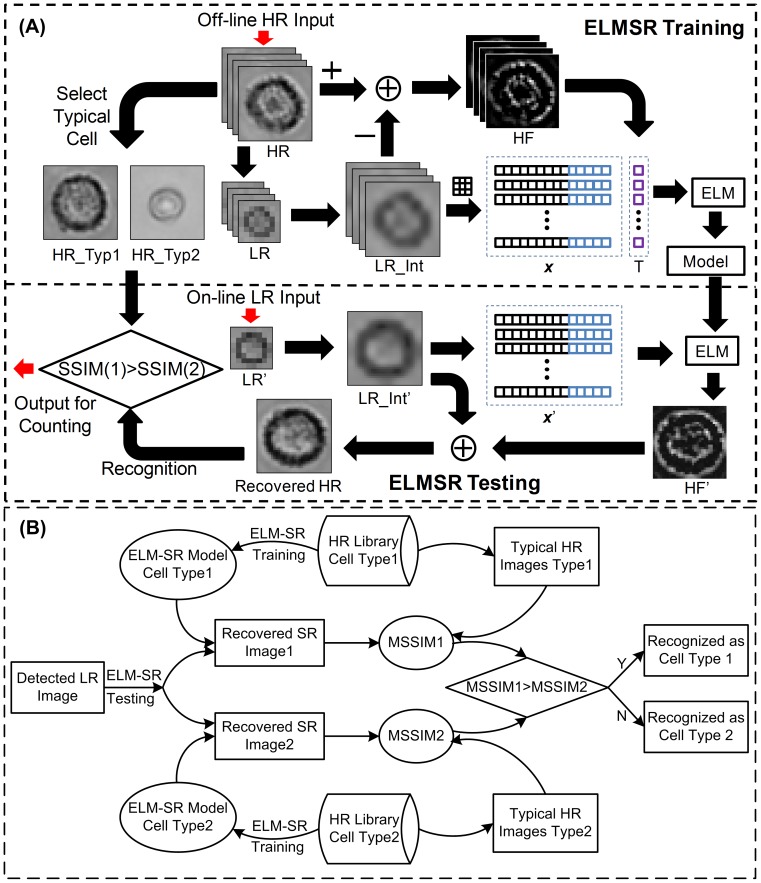
ELM enhanced single-frame super-resolution processing flow. (A) ELM-SR processing flowchart. The training is performed off-line to generate a reference model that can map the interpolated LR images with the HF components from the HR images; and the testing is performed on-line to recover a SR image from the input LR image with the reference model. (B) Flowing cell recognition flowchart. The detected LR image is processed with ELM-SR to obtain SR images according to different off-line trained models. Then, the SR images are compared with typical HR cell images in the library with cell categorized to one type that has the largest MSSIM.

As shown in Fig. 3(A), the ELM-SR includes off-line training and on-line testing. In the training step, a reference model is trained that can map the interpolated LR images with the HF components extracted from the HR images from the training library. The off-line HR training image library is first generated by taking the grayscale HR images of cells with an inverted microscope camera (Olympus IX71, Tokyo, Japan). For one type of cell to generate a HR library, the cell solution is prepared and dropped into the inlet of one microfluidic channel that is bonded on a cover glass. This helps mimic the environment of the microfluidic channel bonded on the CMOS image sensor. Cells suspended in the channel can have different rotations or details in appearance. Thus a number of typical images are taken to generate an HR image library for one cell type under a few appearances. Thereby, the trained reference model is more generic (as a cell neuron) when used for the on-line SR recovery.

In the off-line training step, given the input of HR image *HR_M×N_*, where *M* is the row pixel number and *N* is the column pixel number, a corresponding LR image *LR_m×n_* is first generated through bicuibic down sampling as shown in Fig. 3(A). Note that the down sampling factor is the same as the SR enhancement factor *t*, i.e., *M = m×t*, *N = n×t*. Next, the generated LR image *LR_m×n_* is interpolated back to *LR_Int_M×N_*, which has the same size of *HR_M×N_* but blurred and lack of HF component details. As such, by subtracting the HR image *HR_M×N_* with the interpolated LR image *LR_Int_M×N_*, the HF component *HF_M×N_* is obtained, i.e.,
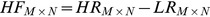
(2)


Based on *p* HF images *HF_M×N_* from the training library, the training targeting value ***T*** is obtained which is a *p•MN×1* row vector of all the pixels intensity values in HF images. Meanwhile, the pixel intensity pattern existed in *LR_Int_M×N_* is extracted by a 3×3 pixel patch *P(i, j)* centered at pixel *(i, j)* of *LR_Int_M×N_* to search through the whole image, where *1≤i≤M–1* and *1≤j≤N–1*. As such, the column vectors extracted from all patches in *p* interpolated images *LR_Int_M×N_* compose the feature matrix ***X***. Thus, the ELM training dataset (***X***, ***T***) is generated.

As such, ELM can take the input (***X***, ***T***) to ELM to calculate a row vector ***β*** containing the weights by
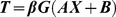
(3)where ***G*** is a sigmoid function, and ***A*** and ***B*** are randomly generated matrix [27]. The training data with ***A***, ***B*** and ***β*** can be used for the ELM-SR reference model.

In the on-line testing step, when a detected LR cell image *LR'_m×n_* is inputted, the corresponding SR image can be recovered using the same ***A***, ***B*** and the trained ***β*** as follows. The resolution of *LR'_m×n_* is first enhanced by *t* times through bicubic interpolation to *LR_Int'_M×N_*_._ The same patch searching method used in the ELM-SR training is applied to extract the feature matrix 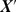 from *LR_Int'_M×N_*. Thus, one can calculate the row vector ***T'*** that includes the recovered HF components *HF'_M×N_* for the input LR image *LR'_m×n_*. As such, the final SR image *SR'_M×N_* is recovered with the sufficient HF details for cell type recognition by 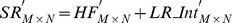
(4)


*B. Flowing Cell Recognition*

Cell type recognition in the developed microfluidic cytometer is performed after recovering the SR image *SR'_M×N_* from the input LR image *LR'_m×n_*. The recognition process is shown in Fig. 2(B). Assume that the samples of interest include two types of cells, two reference models need to be trained for each type of cell. Then when a detected LR cell *LR'_m×n_* is inputted, two SR images, *SR1'_M×N_* and *SR2'_M×N_* can be recovered, each corresponding to one reference model. Afterwards, *SR1'_M×N_* and *SR2'_M×N_* are compared with the typical HR images *HR1_M×N_* and *HR2_M×N_* in the training libraries, where the mean structural similarity (MSSIM/SSIM) index [28] is employed to characterize the similarity. The SSIM is a full reference metric between 0 and 1 to indicate the similarity between one SR image with one distortion-free reference HR image by 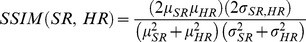
(5)where 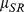 and 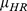 are the means of the SR and HR images, 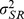 and 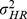 are the variances of the SR and HR images, and 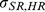 is the covariance of the SR and HR images. It is proven to be consistent with human eye perception compared with traditional metric such as peak signal-to-noise ratio (PSNR) and mean squared error (MSE) [28]. The MSSIM is the average of the SSIMs for one SR image with all the typical HR images,
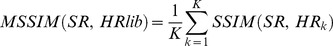
(6)where *K* is the number of typical HR images in the HR training library. For *SR1'_M×N_* and *SR2'_M×N_*, MSSIM1 and MSSIM2 can be calculated. Then we categorize the cell to the type that has the stronger MSSIM.

As such, with the ELM-based single-frame SR processing, the developed microfluidic cytometer can have much better imaging capability to distinguish cell details in the continuously flowing microfluidic channel.

#### Flowing Cell Counting

After recognizing the type for all the detected cells flowing through the channel, the total number of each cell type in the sample of interest can be enumerated. For one cell type, as the cell number in each frame is already known after the temporal-differencing based cell detection step, we subtract the cell number of the current frame with its previous reference frame to obtain a difference value. If this difference is larger than zero, meaning that new cells have flown into the sensor FOV to increase the cell count over the previous frame. As such, we add this difference to the total cell count. By adding all the positive differences after processing the whole series of frames, the total number for one cell type is obtained. For other cell types, the counting procedure is processed in the same and hence their concentration ratio can be eventually obtained.

As such, the detection, recognition, and counting for all the flowing cell types in the testing sample can be achieved, realizing the function of the contact-imaging based microfluidic cytometer.

## Results and Discussion

To evaluate the accuracy of the developed contact-imaging microfluidic cytometer with machine-learning for single-frame super-resolution processing, both of the microbead solution and mixed RBC and tumor cell solution were tested with measurement results compared with a commercially available flow cytometer (BD Accuri C6, NJ, US).

### Counting Performance Characterization

**Figure 4:**
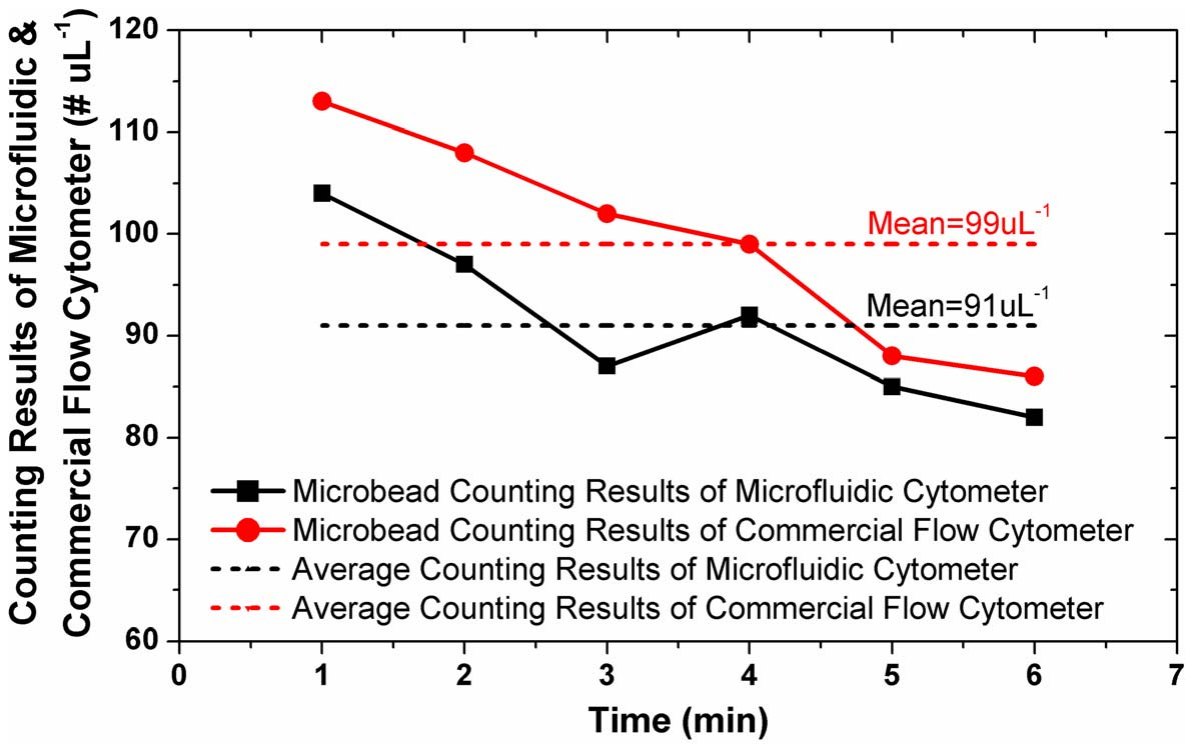
Comparison of concentration measurement results for 6 µm microbead solution between the developed microfluidic cytometer and the commercial flow cytometer. The average counting result of the developed microfluidic cytometer matched well with that of the commercial cytometer with 8% error.

As described in the previous section, the 6 µm polystyrene microbead solution was prepared with a concentration of 100 µL^−1^ measured by the commercial flow cytometer. The 6 µm sample was flushed through the microfluidic channel at a flow rate of 5 µL/min by a syringe pump. Then, a series of 640 frames were captured by the CMOS image sensor for a period of one minute. The total number of microbeads was automatically counted by the developed image processing algorithm. The same process was repeated for 6 minutes, and the measured concentrations of the microbead are shown in Fig. 4. The final microbead concentration is calculated by averaging the counting results of each group, which was 91 uL^−1^ with only 8% error when compared with the result 99 uL^−1^ by the commercial flow cytometer.

**Figure 5:**
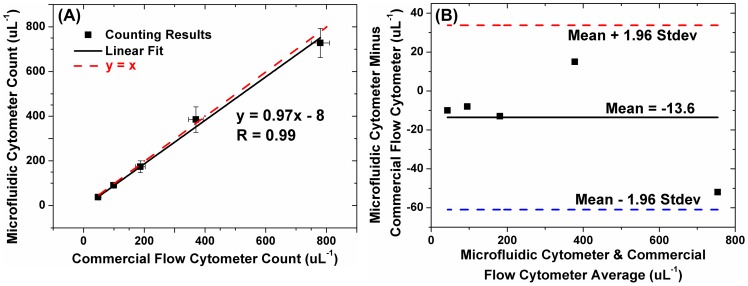
Comparison of counting results of different microbead concentration solutions between the developed microfluidic cytometer and the commercial flow cytometer. (A) Measurement results correlate well between the developed system and the commercial one (y = 0.97x-8, correlation coefficient = 0.996). (B) The Bland-Altman analysis of the measurement results between the developed one and the commercial one show a mean bias of −13.6 uL^−1^, the lower 95% limit of agreement by −61.0 uL^−1^, and the upper 95% limit of agreement by 33.8 uL^−1^.

To further evaluate the developed microfluidic cytometer, five microbead samples of different concentrations, ranging from ∼50 uL^−1^ to ∼800 uL^−1^ were prepared. The flow rate and imaging time were used under the same settings. As shown from Fig. 5(A), the measurement results of the developed microfluidic cytometer correlated well with the commercial flow cytometer with a correlation coefficient of 0.99. Moreover, in order to assess the agreement between the two methods, the Bland-Altman analysis was also performed. As the results shown in Fig. 5(B), a systematic mean bias of −13 cells uL^−1^ was obtained for the developed microfluidic cytometer compared with the commercial flow cytometer. The under counting performance was due to the dead volume in the channel inlet/outlet as well as the cell lost and sedimentation in the tubing.

### Off-line ELM-SR Reference

For the cell recognition, HepG2 and RBC cells were used. The resolution enhancement factor of ×4 was used to improve the LR images. Larger enhancement factor can be selected but at the expense of longer processing time and complexity. Since current LR pixel is 2.2 µm, after SR processing the equivalent pixel size is reduced to 550 nm, enough for the normal cell diagnosis.

**Figure 6:**
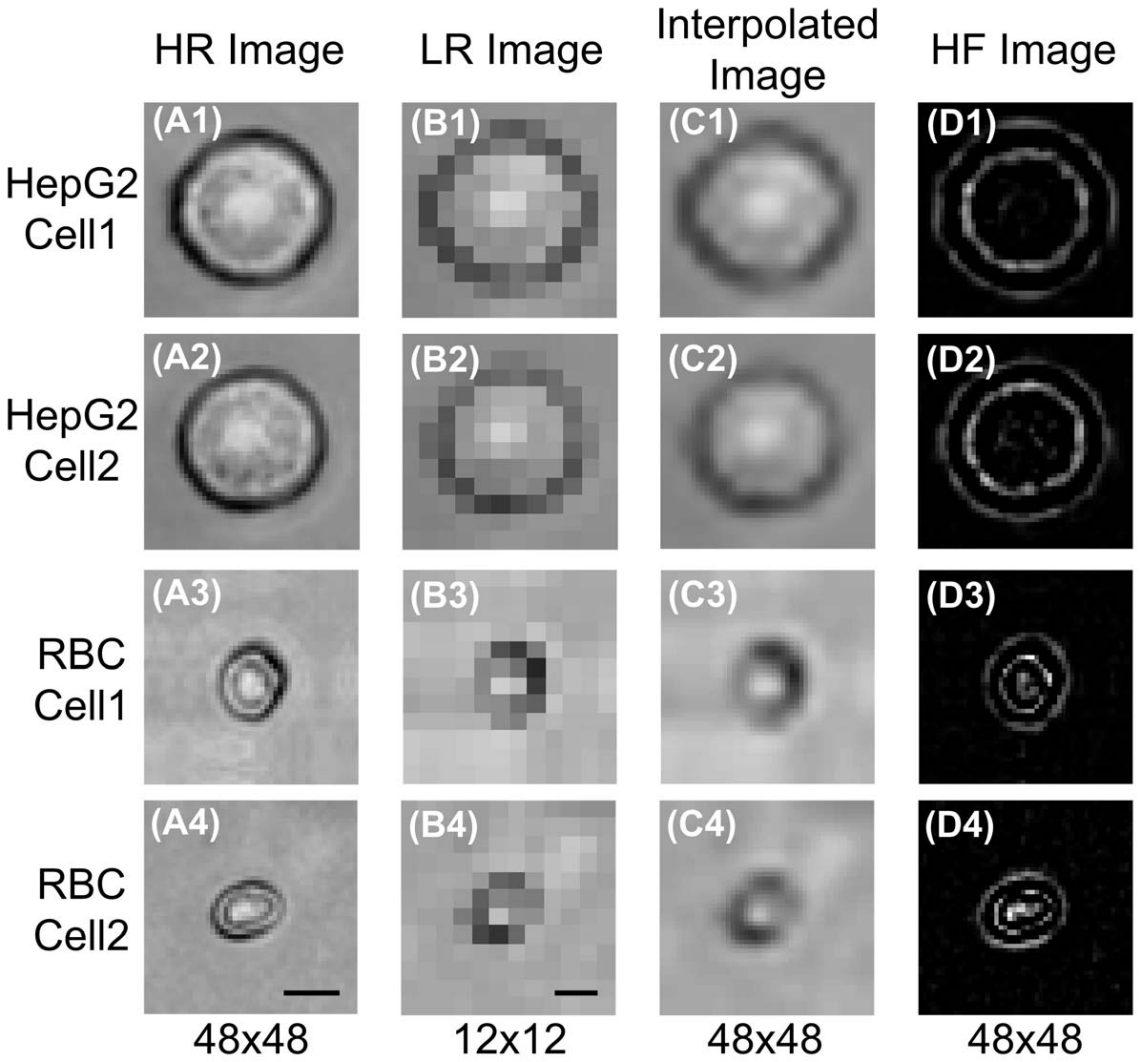
ELM-SR off-line training images for HepG2 and RBC cells. (A) The original HR images for HepG2 cell with two different appearances; and the same for RBC cells. (B) The corresponding LR images. (C) The interpolated images of LR images, which cannot show HF details. (D) The extracted HF components. The scale bar indicates 5 µm.

The off-line training HR image library of HepG2 and RBC was first built. The raw HR images of HepG2 and RBC were taken by the microscope camera at ×40 objective, and saved into the HR image library with the size of 48×48, as shown in Fig. 6 (A1–A4). Then, the corresponding 12×12 LR images were obtained by bicubic down sampling the HR images as shown in Fig. 6 (B1–B4). Next, these LR cell images were interpolated back to the same size of their original HR images, i.e., 48×48. Note that the detailed structures cannot be observed from the interpolated images because the interpolation cannot recover the HF components, as shown in Fig. 6 (C1–C4).

After that, the HF components for each training cell image were obtained by subtracting the interpolated cell images from the original HR images, such as Fig. 6 (D1–D4). As such, the training library was generated and inputted to perform the ELM-SR training and also obtain the reference model (***A***, ***B*** and ***β***). For the current mixed HepG2 and RBC samples, there are 30 HR images selected for each cell type to build the training library.

### On-line ELM-SR Recognition and Counting

**Figure 7:**
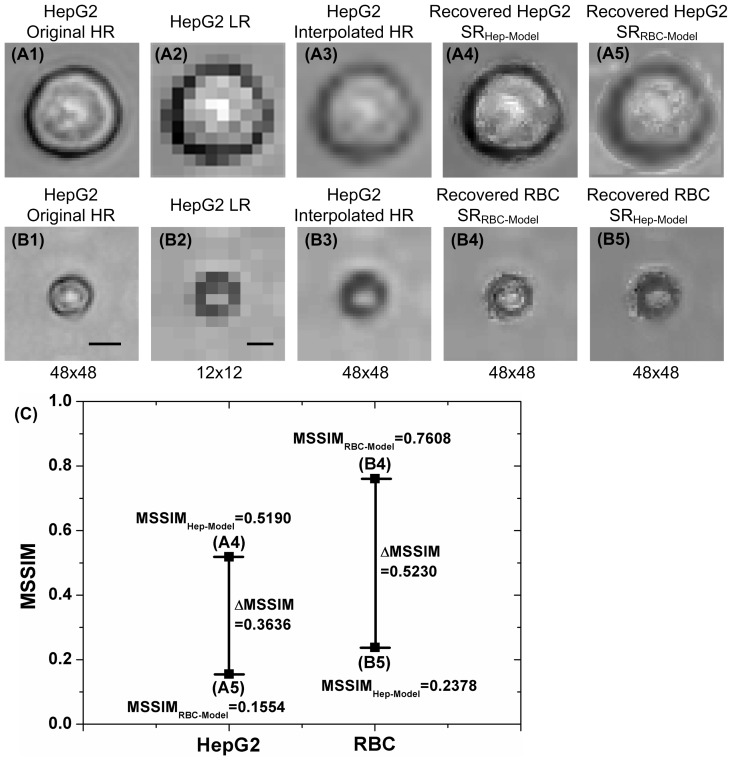
ELM-SR on-line testing results for HepG2 and RBC cells. The resolution is improved by 4× after ELM-SR processing. (A) The HepG2 on-line testing image and the recovered SR image. (B) The RBC on-line testing image and the recovered SR image. (C) The comparison of MSSIM for different SR images obtained under different training models. The detected HepG2 and RBC can be correctly categorized to its type as the SR image recovered by corresponding ELM-SR model produces a larger MSSIM when compared to each cell HR library. The scale bar indicates 5 µm.

After building the off-line training image library and ELM-SR reference models of HepG2 and RBC cells, the on-line ELM-SR processing was performed when an LR image of HepG2 or RBC cell was captured as shown in Fig. 7(A1) and (B1). The recovered SR images using the corresponding trained ELM-SR models are shown in Fig. 7(A4) and (B4), which are defined as HepG2 SR_Hep-Model_ and RBC SR_RBC-Model_. It can be clearly observed that the ELM-SR recovered images show much better cell internal and edge information that the interpolated images of Fig. 7(A3) and (B3) cannot show. The biconcave shape of the SR image of RBC cell can also be observed with sufficient difference from the HepG2 cell.

In addition, the SR image of HepG2 cell recovered by the RBC trained model (HepG2 SR_RBC-Model_) and the SR image of RBC SR cell recovered by the HepG2 trained model (RBC SR_Hep-Model_) are also shown in Fig. 7(A5) and Fig. 7(B5). One can notice large differences when compared with the original HR images.

The MSSIMs with HepG2 library and RBC library are shown in Fig. 7(C). The MSSIMs for HepG2 SR_Hep-Model_ and RBC SR_RBC-Model_ with the HR HepG2 and RBC image libraries are 0.5190 and 0.7608, respectively; and the MSSIMs for HepG2 SR_RBC-Model_ and RBC SR_Hep-Model_ are 0.1554 and 0.2378, respectively. The difference ΔMSSIM of 0.3636 and 0.5230 indicate that the SR image of both HepG2 and RBC have enough MSSIM difference to be distinguished.

**Figure 8:**
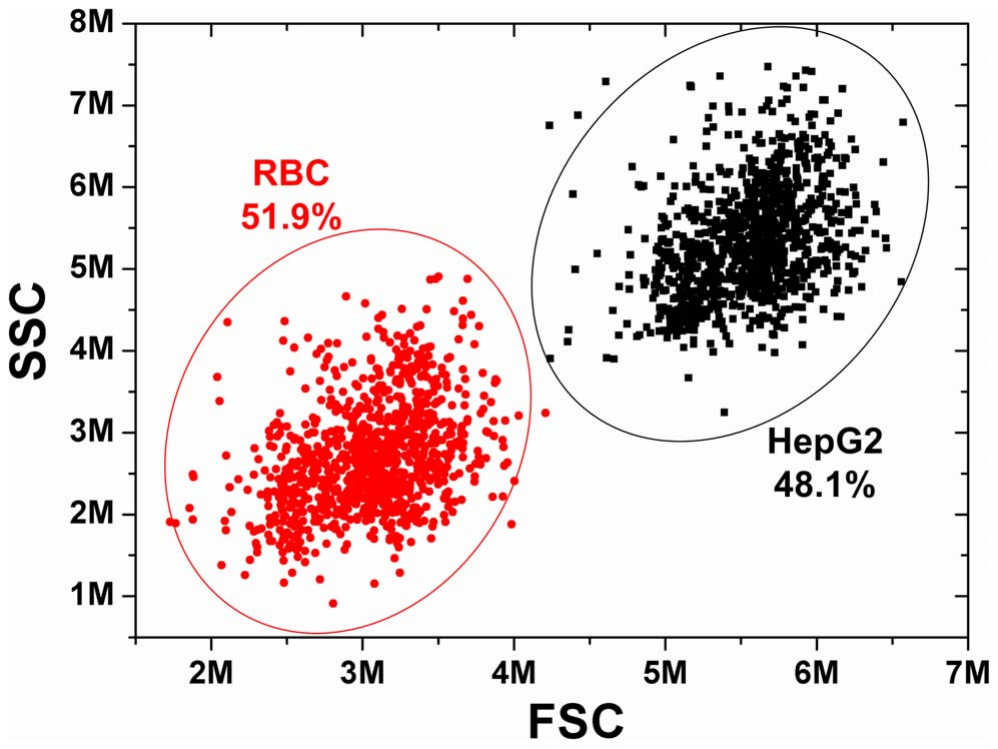
Commercial flow cytometer counting results for the mixed RBC and HepG2 cells. The absolute counts of RBC and HepG2 are 1054 and 978 with the ratio of RBC/HepG2 by 51.9%:48.1% = 1.08: 1.

Furthermore, the ELM-SR was applied to distinguish different flowing cell types when the cell count of each type can be obtained. The ratio between RBC and HepG2 cells was prepared and measured by the commercial flow cytometer with the ratio of 1.08∶1 (51.9%: 48.1%) as indicated in Fig. 8. Then, the sample was tested using the developed microfluidic cytometer at a flow rate of 5 µL/min. As shown in Table 1, the sample was tested for 6 groups, each group for one minute. The mean RBC/HepG2 ratio is 52.60%:47.40% = 1.11∶1 with the coefficients of variation (CV) of 0.10, which matched well with the commercial flow cytometer result (1.08∶1). The CV is lower than many other reported microfluidic cytometers (>15%) [35]. Based on the current sample concentration, the average throughput was 3080 min^−1^. Although the throughput is relatively low from the commercial flow cytometry standard, it can be further improved by increasing the sample concentration and flow rate. Moreover, the continuous microfluidic flow developed in this paper can enable larger volume of sample solution to be examined in each test when compared with the drop and flow method in [17]–[18].

**Table 1:** Measured RBC and HepG2 counting results of the developed microfluidic cytometer with ELM-SR based cell recognition.

Group	RBC (#μL^−1^)	HepG2 (#μL^−1^)	RBC/HepG2
1	239 (54.32%)	201 (45.68%)	1.19
2	338 (50.22%)	335 (49.78%)	1.01
3	260 (53.72%)	224 (46.28%)	1.06
4	435 (52.98%)	386 (47.02%)	1.12
5	340 (55.74%)	270 (44.26%)	1.26
6	334 (49.85%)	336 (50.15%)	0.99
Mean	324 (52.60%)	292 (47.40%)	1.11
Stdev	70	72	0.11
CV	0.22	0.25	0.10

### Discussions

The biological cells are suspended in the fluid when they are flowing by the CMOS sensor. In addition, there is a 6 µm thick PDMS layer coated on the sensor for chip bonding. As such, contact imaging in this paper thereby just emphasizes a close distance between the cell and the CMOS sensor as compared to the conventional one using optical microscopy. It is always desirable to minimize the distance between the cells and the sensor in order to improve the image contrast as well as resolution [23], [36]. However, the physical distance is the fundamental limitation of one contact imaging system with poor resolution. Therefore, considering this first-priority limitation, we have developed an ELM-SR based SR method to recover the low resolution of the contact images of cells.

Furthermore, different from the commercial flow cytometry that can measure FSC and SSC signals, our contact-imaging based cytometer has only one photo detector at the bottom, i.e., the CMOS image sensor. Thus, it can only capture the projected images with light source illuminating from above, similar to the FSC. Meanwhile, the illumination light beams can be arranged with different angles of incidence [37]. When the angle of incidence increases to 90°, the projected images on the CMOS sensor will be equivalent to SSC. Such a design would furnish another strong capability of the proposed contact-imaging based cytometer.

In addition, as for the choice of samples, RBCs and HepG2 tumor cells are among the most common cell types that commercial flow cytometers or other cell counting systems usually deal with. As a preliminary study, we used our prototype to analyse and categorize these two common types of cells into their respective groups according to the notably improved image resolution, which cannot be achieved by the conventional on-chip contact imaging system [36]. In the future follow-up studies, we will further improve this platform on different cell groups with more delicate differences in size and other cellular properties.

## Conclusions

With the use of extreme learning machine for single-frame super-resolution processing, one prototype of contact-imaging based microfluidic cytometer is demonstrated for cell recognition and counting. The developed system resolves the resolution limitation of contact imaging by on-line image recognition based super-resolution processing, which enables continuous high throughput flowing cell recognition and counting. The developed system is validated with comparison to the commercial flow cytometer. The measured results show that the developed system can reach less than 8% error for counting absolute number of microbeads, and can also recognize cell ratio by 0.10 coefficient of variation for the RBC and HepG2 cells in a mixed solution.
